# Transcriptomic Analysis of Young and Old Erythrocytes of Fish

**DOI:** 10.3389/fphys.2017.01046

**Published:** 2017-12-12

**Authors:** Miriam Götting, Mikko J. Nikinmaa

**Affiliations:** Laboratory of Animal Physiology, Department of Biology, University of Turku, Turku, Finland

**Keywords:** red blood cells, aging, transcriptome, fish, lifespan, RNA-seq

## Abstract

Understanding gene expression changes over the lifespan of cells is of fundamental interest and gives important insights into processes related to maturation and aging. This study was undertaken to understand the global transcriptome changes associated with aging in fish erythrocytes. Fish erythrocytes retain their nuclei throughout their lifetime and they are transcriptionally and translationally active. However, they lose important functions during their lifespan in the circulation. We separated rainbow trout (*Oncorhynchus mykiss*) erythrocytes into young and old fractions using fixed angle-centrifugation and analyzed transcriptome changes using RNA sequencing (RNA-seq) technology and quantitative real-time PCR. We found 930 differentially expressed between young and old erythrocyte fractions; 889 of these showed higher transcript levels in young, while only 34 protein-coding genes had higher transcript levels in old erythrocytes. In particular genes involved in ion binding, signal transduction, membrane transport, and those encoding various enzyme classes are affected in old erythrocytes. The transcripts with higher levels in old erythrocytes were associated with seven different GO terms within biological processes and nine within molecular functions and cellular components, respectively. Our study furthermore found several highly abundant transcripts as well as a number of differentially expressed genes (DEGs) for which the protein products are currently not known revealing the gaps of knowledge in most non-mammalian vertebrates. Our data provide the first insight into changes involved in aging on the transcriptional level and thus opens new perspectives for the study of maturation processes in fish erythrocytes.

## Introduction

As in all vertebrates apart from mammals, the erythrocytes in fish are nucleated (Nikinmaa, 1990). Throughout their lifespan, which is between 80 and 500 days (Avery et al., 1992), they appear to be capable of aerobic metabolism, although the respiratory rate decreases with age (Phillips et al., 2000). They are also capable of transcription and translation throughout their lifespan, although, again, the capability decreases with age. The RNA content was significantly lowered by 90% in old erythrocyte fractions while DNA and protein content were unaffected by age (Lund et al., 2000). Young erythrocytes respond much more readily to external stimulation by, e.g., adrenergic drugs than old erythrocytes (Lecklin et al., 2000), and their proportion, which can make up to 50% of the circulating erythrocyte population, increases as a result of temperature stress (Lewis et al., 2012) and changes seasonally (Härdig and Hoglund, 1984). In fish that have recently suffered anemia, the proportion of young RBCs is larger compared to unstressed fish (Lane, 1979).

Associated with the decreased capability to respond to external stimulation, several properties of the erythrocytes are different in young and old erythrocytes. For example, the shape changes from circular to elliptical during maturation (Tavares-Dias, 2006), the membrane of young erythrocytes is more fluid (Lecklin et al., 2000), and old erythrocytes have fewer organelles such as mitochondria (Moyes et al., 2002) and free ribosomes (Phillips et al., 2000).

However, despite these differences in the function and properties of young and old erythrocytes, and although there is a clear difference between the transcriptome of control (11°C) and heat-shocked (1 h at 25°C) erythrocytes (Lewis et al., 2010), quantitative changes in transcription between young and old fish erythrocytes have not been studied earlier. Here we have collected blood from rainbow trout in spring (May) when the proportion of young erythrocytes in the circulation increases with rising ambient water temperature (Härdig and Hoglund, 1984; Alvarez et al., 1994; Houston et al., 1996; Lecklin et al., 2000). We have divided the erythrocytes in young and old cohorts on the basis of the old erythrocytes having higher density than young ones (Lane et al., 1982; Tiano et al., 2000). Since old erythrocytes have higher mean cellular hemoglobin concentration (MCHC) than young ones (Lane et al., 1982), the success of age separation was checked by evaluating MCHC. Thereafter RNA sequencing was carried out and differences in transcriptomes in the young and old cohort evaluated.

## Materials and methods

### Animals and blood sampling procedure

Rainbow trout (*Oncorhynchus mykiss, N* = 5, weight 548.0 ± 70.1 g) were obtained from a commercial hatchery (Finnish Institute for Fisheries and Environment, Parainen, Finland) in May 2016. All procedures were approved by the Finnish Animal Experiment Board (ESAVI/3705/04.10.07/2015). Since the water in the fish tanks of the hatchery is pumped from a nearby bay of the Finnish Archipelago, the water temperature in the tanks follows natural rhythms and is on the rise at this time of the year (K. Malmberg, personal communication). At the date of sampling the water temperature was 14°C.

Fish were netted from the tanks, quickly killed by a blow on the head and blood was sampled by caudal puncture into heparinized syringes, transferred into sterile falcon tubes, and stored on ice. Blood samples were washed three times by repeated re-suspension in rainbow trout saline (128 mM NaCl, 3 mM KCl, 1.5 mM CaCl_2_, 1.5 mM MgCl_2_, 20 mM Tris-HCl, pH 7.6 (Nikinmaa and Jensen, 1992) and centrifugation at 800 × g and 10°C for 3 min to remove buffy coat and white blood cells and stored on ice. The erythrocytes were re-suspended in fresh saline at a hematocrit (Hct) of 18–20% and then stored well-aerated over night at 14°C in cell culture flasks (75 cm^2^) with open caps to allow equilibration. Fish were weighed and their length was measured.

### Erythrocyte age class separation by density centrifugation

Density centrifugation of erythrocyte samples followed essentially the procedure described previously (Murphy, 1973; Speckner et al., 1989; Koldkjaer et al., 2004) with minor modifications. A subsample (referred to as “original blood sample”) was taken and immediately frozen at −80°C. The rest of the erythrocyte sample was once more washed in saline and then adjusted to a hematocrit of ~80% and transferred into polypropylene tubes (length 47 mm, diameter 4 mm, volume 0.5 ml). Tubes were centrifuged in a fixed-angle rotor (45°) at 16,000 × g and 10°C for 30 min. The tubes were cut into three equally sized parts: the top part, containing the youngest and least dense erythrocyte class fraction, the middle part which was discarded, and the lower part, containing the oldest and most dense erythrocytes. Erythrocyte fractions (50 μl) were transferred into new tubes, diluted with 100 μl rainbow trout saline and frozen at −80°C.

The success of age separation was evaluated by determining the mean cellular hemoglobin concentration (MCHC) of both fractions using conventional methods (centrifugation for haematocrit and cyanmethaemoglobin method for hemoglobin concentration; Speckner et al., 1989; Lund et al., 2000). The MCHC of the putatively old erythrocytes was significantly higher than that of the putatively young erythrocytes (*p* = 0.015) indicating that the age separation was successful.

### RNA-Seq of young and old erythrocyte fractions

Erythrocyte samples of different age were then homogenized in TissueLyser (Qiagen, Austin, USA) with two stainless steel beads for 2 × 60 s at 30 Hz and RNA was isolated using NucleoZol (Macherey-Nagel) according to the manufacturer's instructions. RNA was quantified using a NanoDrop 2000 (Thermo Scientific, Bonn, Germany) and quality was checked with a Fragment Analyzer^TM^. Only samples with OD_260/280_ and OD_260/230_ > 1.8 and RIN values higher than 8.7 (range 8.7–9.8) were used in the analyses.

Sequencing libraries were prepared using the Illumina TruSeq Stranded mRNA Sample Preparation Kit and sequenced in two lanes on a HiSeq 3000 instrument at the Finnish Functional Genomics Centre (Turku, Finland) and single-end sequencing chemistry with 50 bp read length.

### Bioinformatic analysis

Base calling on the reads was done using the Bcl2fastq2 software (version 1.8.4). The quality control of raw sequencing reads was performed with FastQC (www.bioinformatics.babraham.ac.uk/projects/fastqc/), and adapters and low quality bases were trimmed by Trimmomatic (Bolger et al., 2014). The read alignment was performed against the reference genome using TopHat v2.1.0 (Kim et al., 2013). We used the Atlantic Salmon genome [ICSASG v2 genome; provided by the International Cooperation to Sequence the Atlantic SalmonGenome (ICSASG; Lien et al., 2016)] as a reference genome because of the status of the higher coverage of the assembly compared to that of the rainbow trout. The number of uniquely aligned reads was between 2.6 and 6.8 M per sample.

The sample correlation values (Spearman's metrics) were between 0.895 and 0.932 (mean 0.910 for young age class, 0.927 for old age class) for all samples.

The gene-wise read counts were normalized using the TMM normalization method of the edgeR R/Bioconductor package before further statistical testing using the Limma R/Bioconductor package. Differentially expressed genes (DEGs) were selected based on a fold change >2 and an FDR (false-discovery-rate) <0.01. Sample pairing (young and old erythrocyte age classes of one individual) was taken into account when building the linear model for statistical testing.

The association between reads and known genes and the number of reads associated with each gene was assessed using the subreads package v1.5.1 (Liao et al., 2014). Differential expression between age classes was analyzed using MA-plots as implemented in DEGseq R package (Wang et al., 2010).

The BLAST2GO software (Götz et al., 2008) was used to predict Gene Ontology (GO) terms for all statistically significant DE genes (FDR < 0.01) and the most abundant genes. Gos are organized hierarchically in terms of biological processes, cellular components and molecular functions. All pseudogenes (labeled with NA) and all tRNAs were removed from the list of DEGs, and the remaining list was subsequently searched against the non-redundant NCBI protein database (NR database) using the BLASTx algorithm (Altschul et al., 1990) with an *E*-value threshold of 10^−5^, and a maximum of 20 “hit” sequences per query was retained. GO annotations were simplified to a smaller set of high-level GO terms using GO slims as implemented in BLAST2GO.

### Differential expression of selected genes using quantitative real-time PCR (qPCR)

qPCR was used to analyze the expression of selected genes on preparations of young and old erythrocytes relative to the original erythrocyte sample (taken before the fractionation procedure) of five individuals. We selected eight genes which on the basis of earlier research have important functions in fish erythrocytes and designed specific primers (Table S1) using the Primer3 software (Koressaar and Remm, 2007; Untergasser et al., 2012) and checked for secondary structures using Beacon Designer Software™ and UNAFold tool (https://eu.idtdna.com/UNAFold). qPCR primers were blasted against the rainbow trout database (NCBI) to ensure specificity.

RNA for qPCR was digested using DNase I (Promega) and 500 ng were reverse transcribed using the RevertAid First Strand Synthesis Kit (Thermo Scientific, Bonn, Germany) using random hexamer primers according to the manufacturer's instructions. cDNA products were amplified in triplicates using the KAPA SYBR® Fast qPCR kit (KapaBiosystems) on a QuantStudio 12K Flex Real-Time PCR System (Thermo Fisher Scientific). Each 10 μL reaction mixture contained 2 μl cDNA template (1:10 or 1:20 dilution) and 0.5 μM of each primer. A two-step cycling protocol was applied: 10 min at 95°C followed by 40 cycles of 15 s at 95°C and 30 s at 60°C and 30 s at 72°C. In a final step, specificity of primers and amplification was evaluated using dissociation curves with a temperature range from 60 to 95°C. All primer pairs generated a single peak in the dissociation curve and PCR efficiency estimated for each primer pair was within the range of 92–105%. Each qPCR plate contained non-template controls to detect potential contamination in reaction mixes. Data were analyzed with the QuantStudio software. Reaction efficiency for each gene was calculated using a standard curve generated from a 1:2 serial dilution of the pooled samples. Standard curve reactions were performed in duplicate. Calculations of relative expression levels with the 2^−ΔΔCT^ method (Livak and Schmittgen, 2001) were done in Microsoft Office Excel and the expression of target genes was normalized against β*-actin* (*act*) expression levels. Stability of β*-actin* expression between young, old and original blood sample was assessed using a one-way repeated measures ANOVA (*p* = 0.106) in Real Statistics Resource Pack software (Release 4.3; www.realstatistics.com; Copyright 2013–2015; Zaiontz, 2015). Data are shown relative to the respective mixed blood samples and log2 transformed. All data are expressed as means ± *SD*. Significant differences were assessed first between raw Ct-values of the young, old and originalsamples, and second between the raw Ct-values of the young and old erythrocyte samples using a one-way repeated measures ANOVA after equal variances (Brown–Forsythe) and normality (Shapiro–Wilk test) of data were tested using the Real Statistics Resource Pack software (Release 4.3; www.realstatistics.com; Copyright 2013–2015) in Excel 2010 (Zaiontz, 2015). In cases where tests for equal variances and normality failed we used a non-parametric test (Friedman's test). *Post-hoc* analyses were done with the Holm-Sidak test. Significance was accepted at the *P* < 0.05 level.

## Results

### Sequencing statistics

In this study, an average number of 12,197,337 reads (mean young erythrocytes: 11,692,319; mean old erythrocytes 12,702,356) were generated. From the 8,273,888 mapped reads (mean young erythrocytes: 8,192,383; mean old erythrocytes 8,355,393), 54% (young erythrocytes: 49%; mean old erythrocytes 59%) could be uniquely mapped to the reference genome. Only uniquely mapped reads (average 4,469,945; mean young erythrocytes 3,956,287; mean old erythrocytes 4,983,602) were used for the downstream analysis, resulting in 51,226 unigenes.

### Most abundant genes in erythrocytes

The 15 most abundant genes (those among the top 10 abundant genes in any sample) account for 9.81–29.35% of all reads (mean young erythrocytes = 23.01 ± 5.76%; mean old erythrocytes = 12.39 ± 1.58%; Table 1). Naturally, various hemoglobin subunit transcripts are among them; *hb*α transcripts accounted for 3.46% and *hbß* for 4.56% of all reads. The products of two abundant transcripts in the list are currently not known.

**Table 1:** Read percentages for the 15 most abundant genes in the erythrocyte samples.

**Genbank ID**	**Gene product**	**Mean**	***SD***	**Min**	**Max**
XR_001327939.1	Uncharacterized LOC106601081, transcript variant X4	3.31	1.72	1.66	5.94
XM_014204292.1	Hemoglobin subunit alpha-like	2.39	1.22	1.00	4.44
XM_014186643.1	Hemoglobin subunit beta-like	1.83	0.82	0.55	3.33
NM_001140225.1	EBV-induced G-protein coupled receptor 2	1.67	0.78	0.51	3.29
NA	NA (pseudogene)	1.95	1.06	0.95	3.67
XM_014146046.1	N-terminal EF-hand calcium-binding protein 1-like	1.18	0.17	0.96	1.50
XM_014204291.1	Hemoglobin subunit beta-like	1.54	0.59	0.85	2.75
XM_014192983.1	Hemoglobin subunit alpha-like	1.07	0.39	0.54	1.62
XM_014203987.1	Hemoglobin subunit beta-1-like	0.86	0.48	0.29	1.66
XM_014201444.1	TBC1 domain family member 15-like	0.31	0.10	0.16	0.45
XM_014204289.1	Hemoglobin subunit beta-1-like	0.51	0.28	0.19	0.99
XM_014168090.1	Stathmin-3-like	0.19	0.23	0.04	0.79
XM_014212639.1	Serine protease 27-like	0.20	0.16	0.07	0.58
NM_001279018.1	Hemoglobin subunit beta-1	0.33	0.17	0.18	0.76
XM_014152620.1	Ribosomal RNA small subunit methyltransferase NEP1-like, transcript variant X1	0.34	0.11	0.18	0.50

For 13 gene IDs GO terms were found by blast search against the NR database. Seven transcripts were associated with the various GO terms related to hemoglobin function (GO:0005833 hemoglobin complex; GO:0005344 oxygen transporter activity; GO:0005506 iron ion binding; GO:0020037 heme binding; GO:0015671 oxygen transport; GO:0019825 oxygen binding). The EBV-induced G-protein coupled receptor 2 (NM_001140225) was assigned to GO terms GO:0016021 (integral component of membrane), GO:0007186 (G-protein coupled receptor signaling pathway), and GO:0002250 (adaptive immune response). The N-terminal EF-hand calcium-binding protein (XM_014146046) was only associated with GO term GO:0005509 (calcium ion binding), while for the transcript of the TBC1 domain family member 15 (XM_014201444) the GO terms GO:0090630 (activation of GTPase activity), GO:0006886 (intracellular protein transport), and GO:0031338 (regulation of vesicle fusion). Stathmin-3 (XM_014168090) was associated with neuron projection development (GO:0031175), cytoplasmic microtubule organization (GO:0031122), regulation of microtubule polymerization or depolymerization (GO:0031110), regulation of GTPase activity (GO:0043087), negative regulation of Rac protein signal transduction (GO:0035021), and microtubule depolymerization (GO:0007019). Serine protease 27 (XM_014212639) is assigned to proteolysis (GO:0006508), and ribosomal RNA small subunit methyltransferase NEP1 (XM_014152620) with methylation (GO:0032259).

### Aging results in lower transcript levels

**Figure 1:**
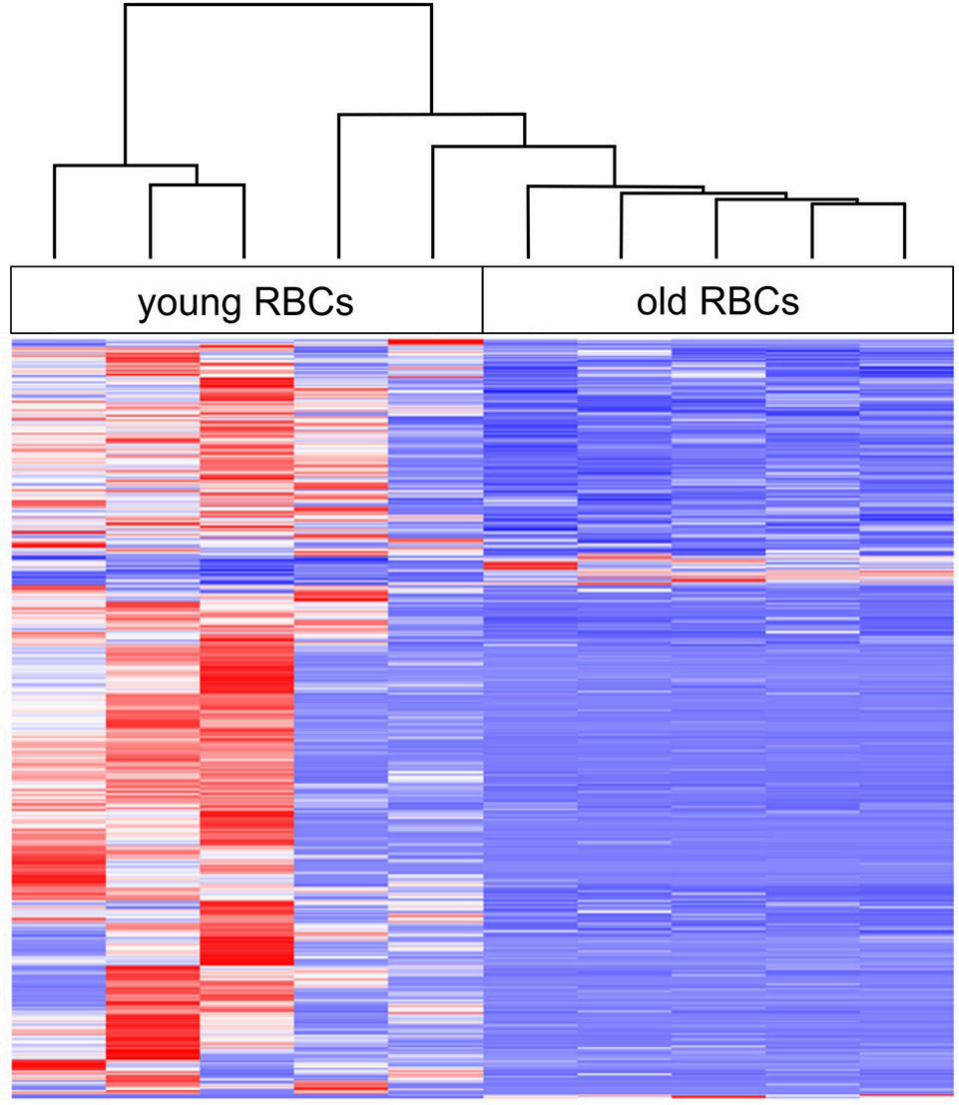
Heat map of age-related changes in gene expression of erythrocytes (RBCs) with hierarchical clustering (complete linkage method with Euclidean distance measure) of all biological replicates. Red indicates highest expression and blue indicates lowest expression.

The DEGs between young and old RBCs were identified by selecting a threshold of > two-fold change in expression level (FDR < 0.01). There was a large shift in transcript abundance between young and old erythrocyte samples (Figure 1). The total number of DEGs was 930; 889 (95.6%) of which show higher transcript levels in young erythrocytes (Figure 1; Table S2), while only 41 (4.4%) have higher transcript levels in old erythrocytes (Figure 1; Table 2). Positive log_2_-fold changes of transcripts in old erythrocytes did not exceed 2.05 (Table 2). The three genes with the highest log_2_-fold changes were the transmembrane protein 215 (XM_014194795), the cancer susceptibility candidate 1, transcript variant X2 (XM_014153053), and the corin, serine peptidase (XM_014206310). Negative log_2_-fold changes of five genes with unknown products were up to −6.5 in old erythrocytes (Table S2).

**Table 2:** List of genes with significantly higher transcript levels in old vs. young erythrocyte fractions.

**Genbank ID**	**Gene product**	**Log2 fold change**	***p*-value FDR adjusted**	**Mean RPKM young fractions**	**Mean RPKM old fractions**
XM_014133936.1	Adaptor protein, phosphotyrosine interaction, PH domain and leucine zipper containing 1, transcript variant X1	1.816	2.52E-08	0.399	1.462
XM_014189649.1	Retinal degeneration 3-like, transcript variant X1	1.418	1.79E-101	81.412	215.543
XM_014210752.1	Protein RD3-like	1.439	1.20E-22	4.092	10.991
XM_014127483.1	Huntingtin-interacting protein 1-related protein-like	1.66	4.04E-08	0.347	1.065
NA	tRNA-Thr	2.061	4.11E-05	0.055	0.982
NM_001141783.1	60S ribosomal protein L4-A	1.52	2.69E-06	0.397	1.068
XM_014194795.1	Transmembrane protein 215	2.048	1.37E-04	0	0.377
XM_014141736.1	Uncharacterized LOC106569954	1.912	1.22E-04	0.066	0.265
XM_014171620.1	Uncharacterized LOC106585433, transcript variant X2	1.896	1.22E-04	0.122	0.549
XM_014153053.1	Cancer susceptibility candidate 1, transcript variant X2	1.927	2.47E-04	0	0.556
NA	tRNA-Thr	1.357	2.91E-06	0.71	1.906
XM_014167525.1	Gap junction delta-2 protein-like	1.32	4.64E-07	4.176	12.823
XM_014206310.1	Corin, serine peptidase	1.92	3.49E-04	0	1.307
XR_001320426.1	Uncharacterized LOC106568984	1.16	1.15E-17	3.978	9.113
XM_014135536.1	SUZ domain-containing protein 1-like	1.651	1.93E-04	0.421	1.377
XM_014200511.1	Protein argonaute-3-like, transcript variant X1	1.165	3.42E-11	1.805	4.151
XM_014181495.1	E3 ubiquitin-protein ligase RNF6-like, transcript variant X2	1.183	1.23E-09	1.051	2.421
XM_014173518.1	Frizzled-5-like	1.502	1.73E-04	0.244	0.716
NM_001195818.1	Sterol regulatory element binding transcription factor 1	1.038	1.49E-23	14.103	28.79
XM_014199009.1	Protein RD3-like	1.476	2.27E-04	0.2	0.55
XM_014191191.1	Autophagy-related protein 9A-like, transcript variant X1	1.148	6.44E-08	2.63	5.709
XM_014212078.1	Transcription factor COE3-like, transcript variant X2	1.173	7.30E-07	1.187	2.613
XM_014212670.1	Alpha-N-acetylgalactosaminide alpha-2,6-sialyltransferase 2-like, transcript variant X1	1.365	1.88E-04	0.122	0.337
XM_014208326.1	Fructose-2,6-bisphosphatase TIGAR B-like	1.146	1.16E-06	1.731	3.853
XM_014194237.1	Mitochondrial import inner membrane translocase subunit tim16-like, transcript variant X2	1.028	6.54E-11	7.329	15.124
XM_014153345.1	G1/S-specific cyclin-D2-like	1.003	1.87E-15	29.248	66.339
XM_014197815.1	Kinesin-like protein KIF3B	1.315	3.18E-04	0.554	1.399
XM_014125557.1	Laminin subunit beta-4-like, transcript variant X2	1.041	2.90E-07	2.665	6.388
XM_014135839.1	Transmembrane protein 9-like, transcript variant X2	1.045	4.37E-07	1.397	2.819
NA	NA	1.265	2.25E-04	0.822	2.033
XR_001322670.1	Uncharacterized LOC106579519	1.236	3.66E-04	0.237	0.606
XM_014205641.1	Transmembrane protein 181, transcript variant X1	1.061	3.58E-05	4.582	9.641
XM_014176665.1	Uncharacterized LOC106588004	1.095	7.95E-05	1.626	3.606
XM_014126943.1	E3 ubiquitin-protein ligase NEDD4-like, transcript variant X4	1.02	1.12E-05	0.677	1.462
XM_014171405.1	Myosin regulatory light chain 2, ventricular/cardiac muscle isoform-like	1.006	1.11E-05	0.76	1.484
XM_014177327.1	Retinoic acid receptor RXR-beta-A, transcript variant X15	1.108	2.23E-04	0.67	1.45
XR_001319380.1	Uncharacterized LOC106563995	1.147	3.61E-04	1.049	2.501
XM_014171986.1	Anthrax toxin receptor 1-like, transcript variant X2	1.027	5.61E-05	1.187	2.49
XM_014140667.1	Peroxiredoxin, transcript variant X1	1.094	3.05E-04	0.748	1.619
NA	tRNA-Met	1.023	3.57E-04	1.903	4.085
XM_014147553.1	leucine rich repeat containing 23, transcript variant X2	1.007	2.77E-04	1.777	3.506

### Functional classification of DEGs

**Figure 2:**
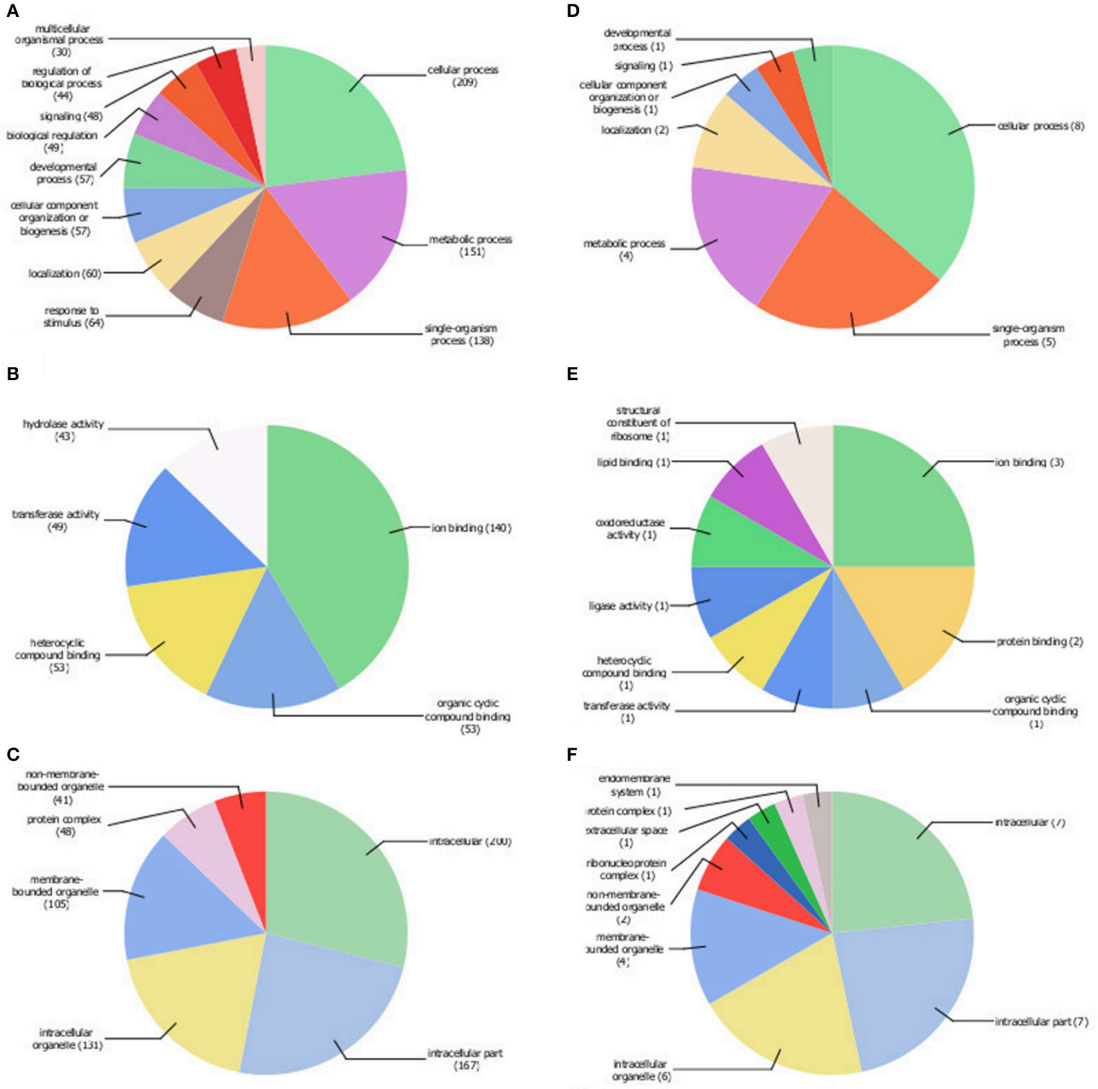
Gene ontology (GO) terms associated with young **(A–C)** and old **(D–F)** erythrocyte age classes. Pie charts were generated at level 2 for biological process **(A,D)**, and level 3 for molecular function **(B,E)** and cellular component **(C,F)** by Blast2GO using 742 transcripts with higher levels in young erythrocytes (Table S2; pie charts **A–C**) and 34 transcripts with higher levels in old erythrocytes (Table 2; pie charts **D–F)** as input.

The gene annotation tool BLAST2GO (Götz et al., 2008), was used to annotate identified DEGs. The input list for the transcripts with higher levels in young erythrocytes contained 742 IDs (Table S2) and for 478 IDs GO annotation terms were found (Figures 2A–C). Eleven terms belonged to the biological process group (Figure 2A), 6 GO terms to the cellular component group (Figure 2C) and 5 GO terms to the molecular function group (Figure 2B). The largest three subcategories in the biological group were cellular processes (209 IDs), metabolic processes (151 IDs) and single-organism processes (138 IDs). In the molecular function category 140 IDs were associated with ion binding, and in cellular component category, 4 enriched GO terms were found: intracellular (200 IDs), intracellular part (167 IDs), membrane-bounded organelle (105 IDs) and intracellular organelle (191 IDs) were enriched terms in the cellular component.

The input list of the transcripts with higher levels in old erythrocytes contained 34 IDs (Table 2), for 18 IDs a total of 25 Gene ontology (GO) annotation terms were identified where 7 were within biological process (Figure 2D), and 9 each in cellular component (Figure 2F) and molecular function (Figure 2E). For 16 IDs no GO terms could be found. GO terms were enriched for cellular process (8 IDs), single-organism process (5 IDs) and metabolic process (4 IDs) in the biological process category. In the metabolic function category, no enriched GO terms were found and with each of the GO terms 1–3 IDs were associated. Intracellular components (7 IDs), membrane-bounded organelle (4 IDs) and intracellular organelle (6 IDs) were enriched terms in the cellular component.

### Expression differences between young and old erythrocyte fractions in relation to the original erythrocyte sample

**Figure 3:**
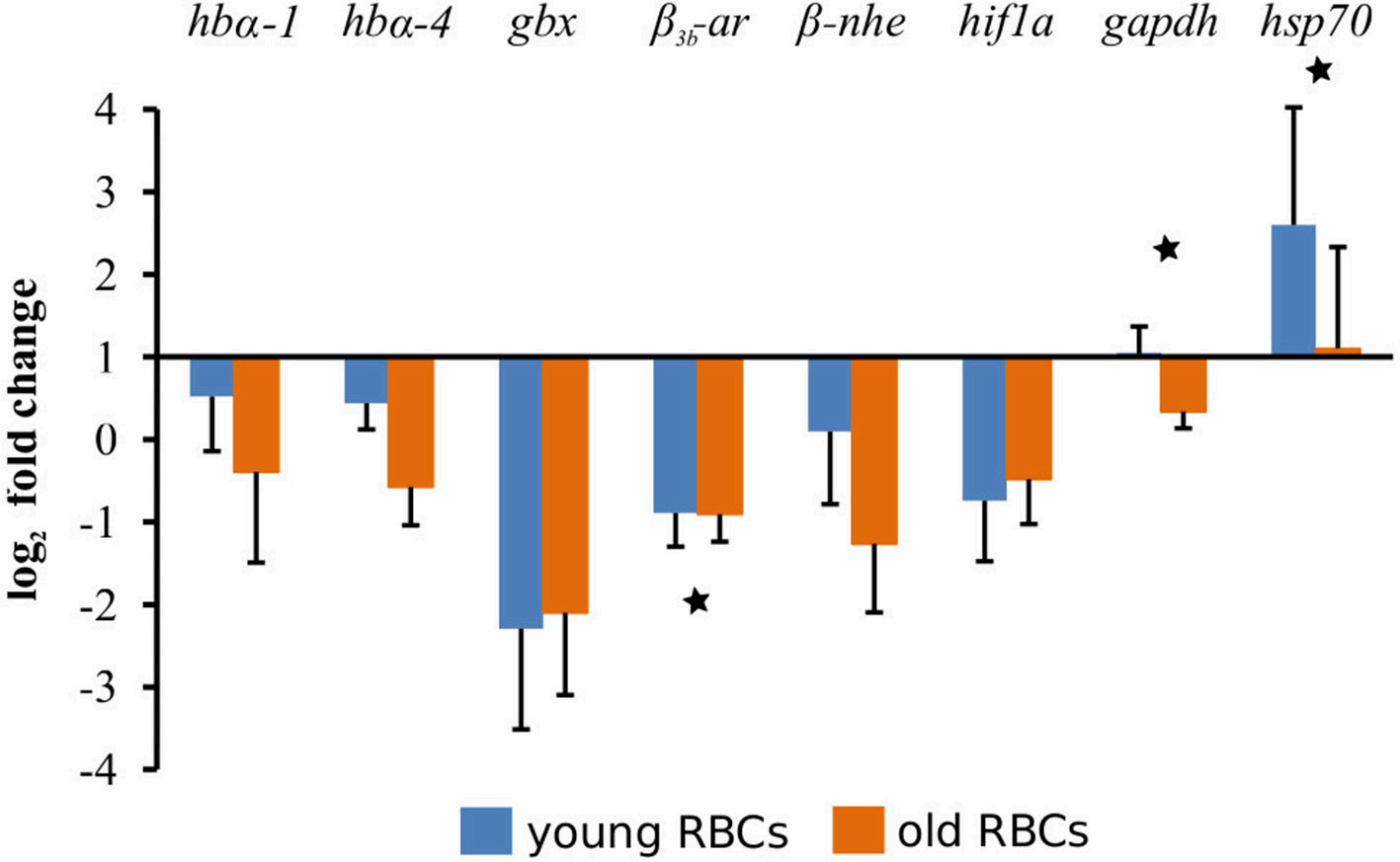
Comparative gene expression levels between young and old erythrocytes (RBCs). Expression of eight target genes was normalized to β*-actin* as a reference gene and then related to the expression of the respective gene in the original erythrocyte sample taken before the fractionation procedure. Gene abbreviations are *hb*α*-1, hemoglobin* α*-1*; *hb*α*-4, hemoglobin* α*-4*; *gbx, globin x1*; β_3*b*_*-ar*, β_3*b*_*-adrenergic receptor*; β*-nhe*, β*-Na*^+^*/H*^+^*-exchanger*; *hif1a, hypoxia inducible factor 1a*; *gapdh, glyceraldehyde-3-phosphate dehydrogenase*; *hsp70, inducible heat shock protein 70 kDa*. Statistically significant differences between means of young and old erythrocyte fractions is shown by ^*^p < *0.05*.

We selected eight genes whose products have important functions in fish erythrocytes for a comparison of expression levels between young and old erythrocyte fractions with the original erythrocyte sample with qPCR. Raw data were first normalized to the expression levels of β*-actin*, and then related to the values of the original erythrocyte sample and finally log_2_ transformed. The levels of expression of the genes are shown in Figure 3. The statistical analyses between young, old and original erythrocyte samples revealed four genes with significant differences: *gbx* (*p* = 0.0408), β_3*b*_*-ar* (*p* = 0.0111), *gapdh* (*p* = 0.0150), and *hsp70* (*p* = 0.007). When only young and old erythrocyte fractions were compared, three of the genes remained significantly different: β_3*b*_*-ar* (*p* = 0.0159), *gapdh* (*p* = 0.0253), and *hsp70* (*p* = 0.0351; Figure 3).

## Discussion

In fish, circulating erythrocytes are a population of different age classes, and their proportion changes with season and as a result of environmental stress (Härdig and Hoglund, 1984; Lewis et al., 2012). Maturation of fish erythrocytes in the circulation is associated with marked changes in many aspects of their physiology. We analyzed these changes on the transcriptomic level in young and old erythrocytes in order to get insights into the yet unknown pathways involved in aging. Changes in cellular transcript levels are the result of a number of tightly controlled processes, such as alteration of transcription rate, transcript stability, or mRNA degradation, and the importance of each of these regulatory steps for the observed changes in transcript levels is not yet known (Götting and Nikinmaa, 2017). Measuring cellular steady-state levels as done by RNA-seq does not take into account alterations in either of these processes (Hayles et al., 2010). Thus, we avoid the terms “up- and downregulated” for changes in transcript levels throughout the manuscript, as it is not known if any of the steps is regulated or just changes.

Our results revealed that aging in fish erythrocytes is accompanied by a decrease in transcript levels of a number of genes. It was shown earlier that the rate of transcription and the cellular RNA content decreases during maturation of nucleated non-mammalian erythrocytes (Grasso et al., 1977; Wiersma and Cox, 1985; Lund et al., 2000). High transcriptional and translational activity is required in young, immature erythrocyte because they gradually undergo changes in cell shape, membrane rigidity and other properties until maturation (e.g., Lecklin et al., 2000; Tavares-Dias, 2006). In contrast, in old, mature erythrocytes the need for gene transcription and protein synthesis is much lower and the necessary machinery, such as ribosomes is very low (Lane et al., 1982; Phillips et al., 2000). A total of 51,226 unigenes could be identified in the present study but we found only 930 (1.8%) DEGs. Most of the DEGs (95.6%) had significantly decreased levels in old erythrocytes and the overall gene expression is much more variable between young erythrocyte samples than between old erythrocyte samples, which appear more homogeneous (Figure 1). The higher transcriptional variability in young erythrocytes is associated with their more active roles in various physiological processes such as in the perception of external stimuli, adaptation and the immune response compared to the terminally differentiated, mature erythrocyte (Lecklin et al., 2000; Koldkjaer et al., 2004; Morera et al., 2011).

The most abundant transcripts in terms of number and expression level belonged to hemoglobin (Hb). Fish exhibit a remarkable multiplicity of Hbs with different functional properties, such as differences in O_2_-affinity and sensitivity to allosteric regulators, which provide an important molecular strategy for adapting to a changing environment (Weber, 1990). During aging some hemoglobin subunit transcripts decrease significantly, while others remain at the same level. It has been reported earlier that Hb accumulates in erythrocytes during maturation and older erythrocytes are tightly packed with Hb (Bastos et al., 1977; Clark, 1988). Older erythrocytes thus serve merely as oxygen transporters. Even if the overall *hb* transcript levels are decreased in older erythrocytes, a long transcript half-life and a high stability of Hb protein ensures adequate synthesis for required function throughout the lifespan in the circulation.

Surprisingly, our study revealed that for two of the 15 most abundant transcripts in the samples the products and thus the function is not known. A blast search revealed a total of 7 transcript variants for the locus LOC106601081, and a blastx search using the unknown protein product of transcript variant 1 (XM_014192985) as query, found no hits. Further studies are required to identify and characterize the function of this transcript.

The second unknown abundant transcript is a pseudogene. A number of pseudogenes (*N* = 1993) were found to be present in the erythrocyte samples but more than half of them were only little expressed. Pseudogene transcripts are very often non-functional copies of real genes which have lost some or all of their functionality by accumulating mutations that disable, e.g., translation. Although several studies discussed the existence of pseuogenes in various organisms, another source of pseudogenes is the read alignment step during RNA-seq data analysis (Tonner et al., 2012).

Other abundant transcripts have functions in the intracellular protein transport and GTPase activation (TBC1 domain family member 15), calcium ion binding (N-terminal EF-hand calcium-binding protein) or the cytoplasmic microtubule organization (Stathmin-3). Furthermore, highly abundant transcripts of the serine protease 27, responsible for proteolysis, and of the ribosomal RNA small subunit methyltransferase NEP1, involved in methylation, were found. Among the genes with abundant levels only the transcripts of genes encoding EBV-induced G-protein coupled receptor 2 are significantly reduced by 88% in old erythrocytes, suggesting an impairment of the G-protein coupled receptor signaling pathway and a massively reduced adaptive immune response in old erythrocytes. An earlier study revealed already that nucleated erythrocytes play an active role in the immune response (Morera et al., 2011), but according to our results this is probably mostly restricted to young erythrocytes.

**Figure 4:**
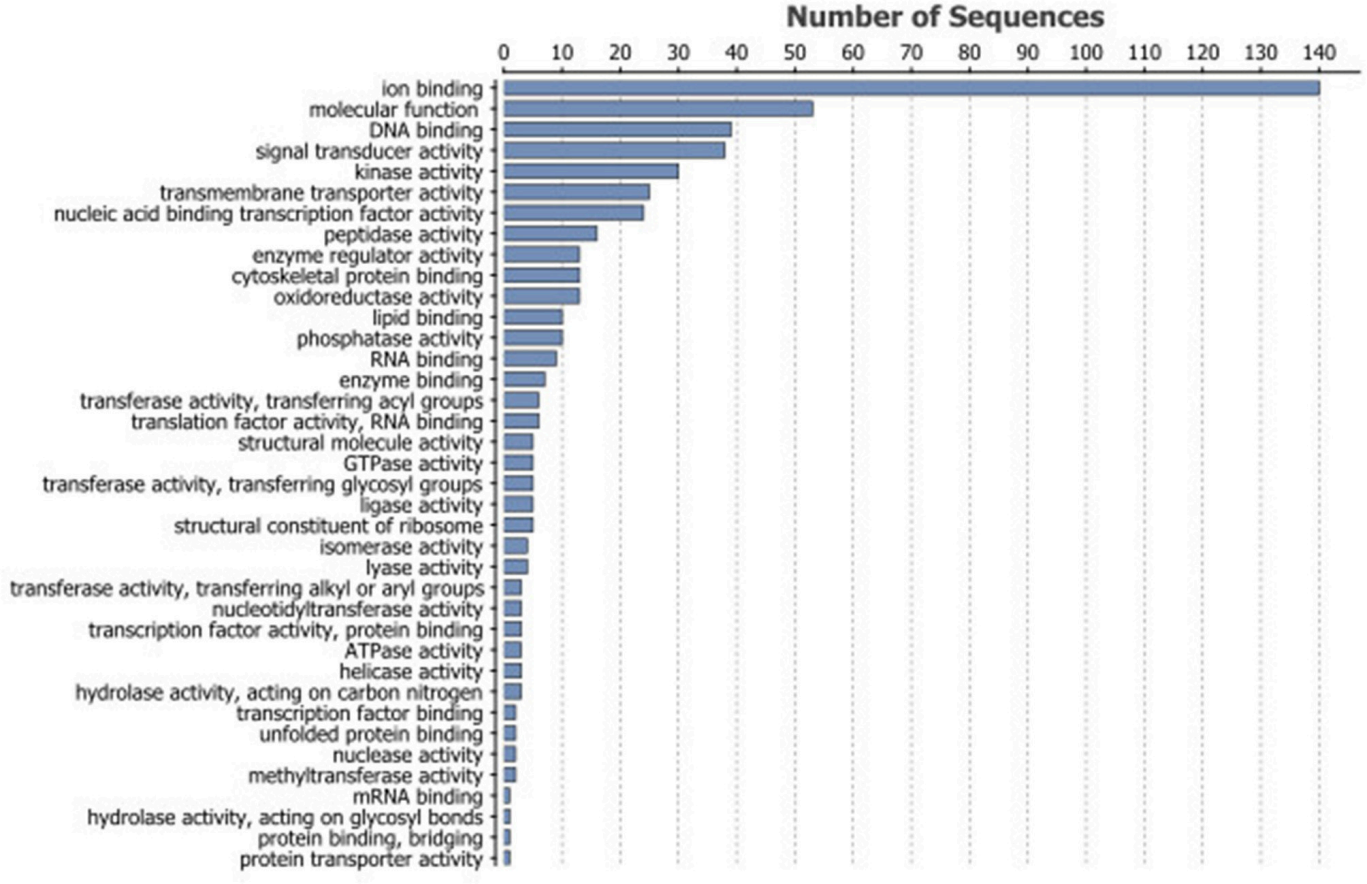
Most frequent GO terms associated with the 742 DEGs with lower transcript levels in old erythrocytes.

Age-dependent decreases in transcript levels of proteins involved in many molecular pathways were found in the present study (Figure 4). In particular genes whose products have functions in ion binding and DNA-binding or in signaling and membrane transport have significantly reduced transcript levels in old erythrocytes. Several enzyme classes (e.g., kinases, oxidoreductases, peptidases) seem to be affected as well. Earlier studies found that mRNA levels of carbonic anhydrase and Cl^−^/HCO_3_-exchanger (AE1) decrease with age (Lund et al., 2000), and also enzyme activity levels of carbonic anhydrase, citrate synthase, cytochrome oxidase and lactate dehydrogenase in rainbow trout RBCs (Lund et al., 2000; Phillips et al., 2000) are lower.

Only a small proportion of the DEGs (*N* = 41; 4.4%) showed higher levels in old erythrocytes. Among them, 34 are protein-coding and only for three the products are currently not known, although their log_2_-fold changes are comparably high. Two of the three genes with the highest log_2_-fold changes are integral components of the membrane. Currently little is known about the transmembrane protein 215 (XM_014194795) and its function. It is conserved among vertebrate species and is expressed in mouse bipolar cells (Park et al., 2017). An attractive speculation is, because of the increase of its transcription in old cells, that it is involved in the age-dependent removal of cells from circulation. For the corin, serine peptidase (XM_014206310; Table 1) the blastx search against the NR database revealed a hit of the atrial natriuretic peptide-converting enzyme of *Oreochromis niloticus*, which is involved in proteolysis and serine-type endopeptidase activity. For the cancer susceptibility candidate 1, transcript variant X2 (XM_014153053) no blastx hits and thus no annotations were found. For almost half of the DEGs with higher levels in old erythrocytes (*N* = 16; 47%) no GO terms could be retrieved from the databases revealing the gaps in knowledge for non-mammalian non-model organisms.

Using quantitative real-time PCR we analyzed the changes of eight genes, which have important functions in fish erythrocytes, in young and old fractions and compared them to the original erythrocyte sample. Four transcripts (β_3*b*_*-ar, gapdh, gbx*, and *hsp70*) had significantly different levels between the young, old and the original sample. When only young and old fractions were compared, three transcripts were found to be significantly different (β_3*b*_*-ar, gapdh*, and *hsp70*; Figure 3), which are not among the DEGs in the RNA-seq results (Table S2). This is due to the different statistical cutoffs applied for the RNA-seq data (FDR adjusted *p*-value, see Materials and Methods section) vs. a significance level of p < 0.05 used in the qPCR. When using the not-adjusted *T*-test *p*-value for the RNA-seq data, β_3*b*_*-ar* and *gapdh* also have significant different levels in young and old fractions (*p* = 0.02 and *p* = 0.03, respectively), while *hsp70* remained non-significant (*p* = 0.05). The other transcripts (*hb*α*-1, hb*α*-4, hif1a*, and β*-nhe*) studied showed the same results as the RNA-seq data and were not different between age class fractions.

The higher β_3*b*_*-ar* transcript levels in young fractions and the decrease with age is in accordance with earlier reported results and explains why the response of older, mature erythrocytes to external stimuli such as catecholamines is impaired (Cossins and Richardson, 1985; Lecklin et al., 2000). β_3*b*_*-ar* transcript levels in rainbow trout vary with season, showing high levels in summer (May to October) and low levels in winter (December to May) (Koldkjaer et al., 2004). Although β_3*b*_*-ar* mRNA was not compared between young and old erythrocyte fractions by Koldkjaer et al. (2004), the observed increase in mRNA can be mainly attributed to the increased proportion of young erythrocytes in the in spring.

Older erythrocytes have a lower ability to produce stress proteins during the recovery period after heat shock (Lund et al., 2000). We found significantly higher transcript levels for the heat shock protein 70 kDa in young erythrocytes in both qPCR and RNA-seq data, but transcript levels of HSF-1 and Hsc71 were unaffected by cell age. This is in contrast to earlier study reporting lower transcript levels for the heat shock cognate 71 kDa (Hsc71) and the heat shock factor (HSF) in old erythrocytes compared to younger ones (Lund et al., 2000).

## Conclusion

The mechanisms and characteristics which finally promote the removal of erythrocytes from the circulation and their destruction are still not known in fish. We found only a small proportion of DEGs between young and old fractions suggesting that even rather subtle changes in gene expression can have significant impact on a wide variety of molecular functions and the cell fate. The diversity of transcripts found in both erythrocyte age classes implies that the physiological roles of nucleated erythrocytes go far beyond the already known functions in oxygen transport and immune response. Hence, our data provide a rich resource for future investigations on the regulation of aging in fish and in particular fish erythrocytes and help to uncover additional physiological functions.

## Data deposition

The RNA-seq data have been deposited at the NCBI Gene Expression Omnibus (GEO) with the accession number GSE106570.

## Author contributions

MG and MN: conceived the study and wrote the paper; MG: performed the experiments and analyzed the data.

### Conflict of interest statement

The authors declare that the research was conducted in the absence of any commercial or financial relationships that could be construed as a potential conflict of interest.

## Supplementary material

Table S1 Primers used in qPCR.

Table S2 List of genes with significantly higher transcript levels in young vs. old erythrocyte fractions.
