# Supplementary material for: Transcriptomic Analysis of Young and Old Erythrocytes of Fish
Source: Front Physiol. 2017 Dec 12;8:1046. doi: 10.3389/fphys.2017.01046 (PMC5732906; doi:10.3389/fphys.2017.01046)
Supplement: Table S1 — Primers used in qPCR. [file DataSheet1.DOCX]

**Table S1. Primers used in qPCR.**

| Gene | Gene symbol | Primer for (5'-3')  Primer rev (5'-3') | Amplicon size (bp) | Efficiency | Genbank accession |
| --- | --- | --- | --- | --- | --- |
| *β-actin* | *β-actin* | TCAACCCCAAAGCCAACAGG  AGAGGCGTACAGGGACAACA | 107 | 2.00 | NM_001124235 |
| *hemoglobin α -1* | *hbα-1* | GTCTGACAGCAAAGGACAAATCT  CTCAGCACCGACGACATCT | 80 | 1.95 | NM_001124551 |
| *hemoglobin α -4* | *hbα-4* | AGGACAAAGCTAACGTGAAGGC  AGACTACGAGCATCCTGGAAAGA | 93 | 2.03 | XM_021557875 |
| *globin x1* | *gbx1* | ACAACGCCCCACCCAAATAC  TCCATGCCTCCTCAAGCTCA | 105 | 2.00 | Götting & Nikinmaa (2017) |
| *β-Na^+^/H^+^-exchanger* | *β-nhe* | AGGAGTTGAGGGTGTCTGTGG  TTCACGTAGGTCTTGTTGAAGCG | 80 | 2.00 | M94581 |
| *β_3b_-adrenergic receptor* | *β_3b_-ar* | TCGTGTGGCTATGAGGAAGACT  TGCCTGCTGTGACTTTATGACC | 121 | 1.95 | NM_001124452 |
| *hypoxia inducible factor 1a* | *hif1a* | CAGCCCCAGTGTGTTGTGTG  GCCTCATATCCTCCGTCTGCT | 94 | 2.00 | AF304864 |
| *glyceraldehyde-3-phosphate dehydrogenase* | *gapdh* | CCTGGTATGACAATGAGTTTGG GCATGTACAGCAACAGGTCAG | 60 | 1.99 | XM_021596797 |
| *inducible heat shock protein 70 kDa* | *hsp70* | TGGGCTGAATGTGCTGAGGA  CGTTGCGTTCCCTGGACTTG | 89 | 1.94 | AB062281 |
